# Association of Increased Serum S100B Levels With High School Football Subconcussive Head Impacts

**DOI:** 10.3389/fneur.2019.00327

**Published:** 2019-04-05

**Authors:** Steven W. Zonner, Keisuke Ejima, Zachary W. Bevilacqua, Megan E. Huibregtse, Carmen Charleston, Ciara Fulgar, Keisuke Kawata

**Affiliations:** ^1^Department of Sports Medicine, Washington Township Medical Foundation, Fremont, CA, United States; ^2^Department of Epidemiology and Biostatistics, School of Public Health, Indiana University, Bloomington, IN, United States; ^3^Department of Kinesiology, School of Public Health, Indiana University, Bloomington, IN, United States; ^4^Division of Washington Sports Medicine, Irvington High School, Fremont, CA, United States; ^5^Center for Health and the Environment, University of California, Davis, Davis, CA, United States; ^6^Program in Neuroscience, College of Arts and Sciences, Indiana University, Bloomington, IN, United States

**Keywords:** subconcussion, brain injury, blood biomarker, football, youth, concussion, head impact kinematics, astrocyte

## Abstract

Astrocyte-enriched marker, S100B, shows promise for gauging the severity of acute brain trauma, and understanding subconcussive effects will advance its utility in tracking real-time acute brain damage. The aim of the study was to investigate whether serum S100B elevations were associated with frequency and magnitude of subconcussive head impacts in adolescents. This prospective cohort study of 17 high-school football players consisted of the following 12 time points: pre-season baseline, 5 in-season pre-post games, and post-season. A sensor-installed mouthguard recorded the number of head impacts, peak linear (PLA) and peak rotational (PRA) head accelerations from every practice and game. During the 5 games, players wore chest-strap heart-rate monitors to estimate players' excess post-exercise oxygen consumption (EPOC), accounting for physical exertion effects. At each time point, blood samples were obtained and assessed for S100B and creatine kinase levels to account for astrocyte damage/activation and muscle damage, respectively. Using *k*-means clustering on the impact data, players were categorized into high- or low-impact group. Two players withdrew during the first month of the study. A total of 156 blood samples from 15 players were assessed for S100B and creatine kinase levels and included in the analysis. A median value of 596 head impacts from 15 players were recorded during all practices and games in a season. S100B levels were significantly elevated in all post-game measures compared with the respective pre-game values (median-increase, 0.022 μg/L; interquartile-range, 0.011–0.043 μg/L, *p* < 0.05 for all games). Greater acute S100B increases were significantly associated with greater impact frequency, sum of PLA and PRA, with negligible contributions from physical exertion and muscle damage effects. The high-impact group exhibited greater increases in serum S100B levels at post-games than the low-impact group (high vs. low, 0.043 ± 0.035 μg/L vs. 0.019 ± 0.017 μg/L, *p* = 0.002). The degree of acute S100B increases was correlated with subconcussive head impact exposure, suggesting that acute astrocyte damage may be induced in an impact-dependent manner. Acute changes in serum S100B levels may become a useful tool in monitoring real-time brain damage in sports.

## Introduction

Annually, approximately 2.5 million high school and college athletes engage in contact sports that frequently induce rapid acceleration-deceleration of the body and head (1). These forces, without eliciting outward clinical symptoms of concussion, are referred to as subconcussive impacts, and contact-sport athletes reportedly endure several hundreds to a thousand subconcussive impacts per season (2–4). Long-term, repeated exposure to subconcussive head impacts has been suggested as a key factor for development of chronic traumatic encephalopathy (CTE) (5–9).

In the quest of establishing a highly sensitive and specific objective blood biomarker for brain injury, S100B has been extensively studied in different types and severities of traumatic brain injury (TBI). S100B, a member of the S100 family regulating intracellular calcium levels, is preferentially expressed in astrocytes and increases its expression after astrocyte damage (10). The degree of S100B increase in the blood has shown to reflect the severity of brain damage (11–14). In 2013, Marchi et al. for the first time suggested that serum S100B levels increased in concert with frequency of subconcussive head impacts in high school football players, which were counted through film analysis (15). This finding was subsequently corroborated by Kawata and his colleagues who employed a sensor-installed mouthguard to objectively record frequency and magnitude of head impacts during Division I college football practices (16–18). They found a robust positive correlation between head impact kinematics and changes in S100B levels before and after practices. However, there is a line of research suggesting potential modulating effects from physical exertion and muscle damage on circulating S100B levels (19, 20). For instance, some data sets have failed to differentiate athletes with concussion from extraneous exercise groups (20–22). This may be due to increases in S100B levels following strenuous sports activity, as additional studies have detected elevated S100B concentrations in the serum of professional athletes and age-matched controls post-exercise (19, 23). Moreover, musculoskeletal damage from orthopedic injury may have a significant modulating effect on circulating S100B levels (21, 24, 25). Patients with multi-trauma (orthopedic injury plus head injury) have shown a significantly higher level of S100B than that of isolated head injury and orthopedic injury without head injury, suggesting that S100B derived from muscle damage may influence S100B expressions in the blood.

Since the aforementioned two subconcussion studies (15, 17) failed to account for the potential confounding factors, the relationship between subconcussive head impacts and astrocyte damage, as reflected in the increased serum S100B levels, remains inconclusive. Therefore, in this longitudinal, prospective cohort investigation of high-school football players, we aimed to test whether acute increases in serum S100B would be associated with subconcussive head impact frequency and magnitude, measured by a sensor-installed Vector mouthguard. We also examined if the degree of association would be strengthened after accounting for physical exertion effects as reflected by an excess post-exercise oxygen consumption (EPOC) and for muscle damage effects as measured by serum creatine kinase-skeletal muscle isoenzyme (CK-MM) levels (26, 27). We further clustered the impact data into a low or high impact group and tested whether serum S100B levels would be elevated in those with repeated subconcussive impacts compared to the counterpart with lesser impact exposure. Lastly, we aimed to determine whether there would be chronic elevation of S100B as compared in pre- and post-season levels.

## Materials and Methods

### Participants

Seventeen high school football players at Irvington High School of the National Federation of State High School Associations (NFHS) volunteered for this study. The study was conducted during the 2017 football season, including a preseason physical examination on July 18, 2017, 5 in-season games (September 22, October 16, 20, and 28, and November 4, 2017), and postseason follow-up (December 1, 2017: Figure 1). Those 5 games were randomly selected a priori using a randomization protocol in the R statistical software. None of the 17 players were diagnosed with a concussion during the study period as confirmed by team athletic trainer and physician. Inclusion criterion was being an active football team member. Exclusion criteria included a history of head and neck injury in the previous 1 year or neurological disorders. Since our primary analysis was to correlate acute pre-post changes in S100B with head impact kinematic data, only completed samples from both pre- and post-game were included in the analysis. All participants and their legal guardians gave written informed consent, and the Washington Hospital Healthcare System Institutional Review Board approved the study.

**Figure 1:**
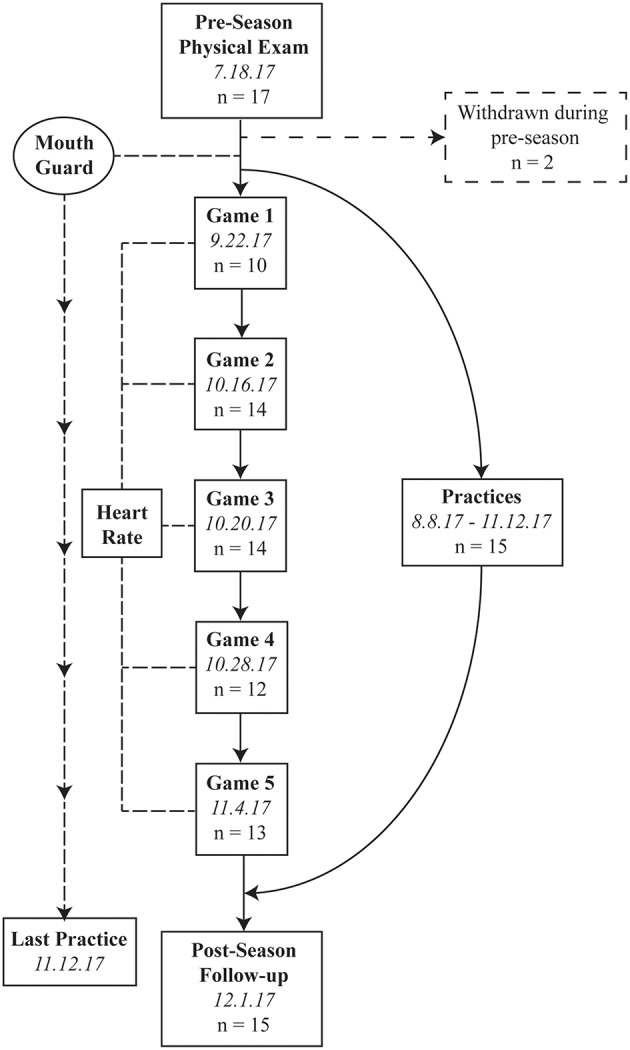
Study flow chart.

### Study Procedures

During the preseason physical examination, participants were custom-fitted with the Vector mouthguard (Athlete Intelligence, Kirkland, WA) that measured the number of hits and magnitude of head linear and rotational acceleration. Players wore the mouthguard for all practices and games from the beginning of the training summer camp (August 8, 2017) to the end of the season (November 11, 2017). Players were also fitted with a wireless chest-strap heart-rate monitor (Firstbeat Technologies, Jyväskylä, Finland) to record variability of heart rate during the 5 games. During the preseason baseline assessment, self-reported demographic information (age, height, weight, history of concussion, and years of American football experience) and blood samples were collected. There were no practices or games between the final day of the season (November 11, 2017) and post-season data collection (December 1, 2017).

### Head Impact Measurement

This study used an instrumented Vector mouthguard for measuring linear and rotational head kinematics during impact as previously described (16, 17, 28). The mouthguard employs a triaxial accelerometer (ADXL377, Analog Devices, Norwood, MA) with 200 g maximum per axis to sense linear acceleration. For rotational kinematics, a triaxial gyroscope (L3GD20H, ST Microelectrics, Geneva, Switzerland) was employed. When a preset threshold for a peak linear acceleration (PLA) magnitude exceeded 10.0 g, a standard hit duration of 93.75 ms of all impact data were transmitted wirelessly through the antenna transmitter to the sideline antenna and computer, then stored on a secure internet database. From raw impact data extracted from the server, the number of hits, PLA, and peak rotational acceleration (PRA) were used for further analyses. Four observations were consistent outliers on the number of hits, sum of PLA, and sum of PRA, exceeding at least 5.5 standard deviations above the mean. These observations were excluded from analysis (<2.5% of all data).

### Blood Collection and Assessment for S100B and Creatine Kinase

Blood samples were obtained from 12 time points. For the 5 game days, pre-game blood samples were collected at 4–5 h prior to competition to ensure no effect from pre-game warm up, and post-game blood samples were collected within 1 h after the games. At each time point, 4 ml of venous blood samples were collected into red-cap serum vacutainer sterile tubes (BD Bioscience). Blood samples were allowed to clot at room temperature for a minimum of 30 min. Serum was separated by centrifugation (1,500 x g, 15 min) and stored at −80°C until analysis. S100B and CK-MM measurements were performed using sandwich-based enzyme-linked immunosorbent assay (ELISA) kits (S100B: EMD Millipore, Billerica, MA; CK-MM, LifeSpan Biosciences Inc., Seattle, WA). Serum levels of CK-MM have been accepted as a surrogate marker of muscle damage (26). The lowest detection limit of the assay is 0.0028 μg/L for S100B and 0.94 μg/L for CK-MM. The highest detection limit of the assay is 2 μg/L for S100B and 100 μg/L for CK-MMwith a typical intra-assay precision of <2.9 and <6.8% and an inter-assay precision of <1.9 and <5.3% for S100B and CK-MM, respectively. Fifty microliters (S100B) and 100 μl (CK-MM) of serum samples were loaded in duplicate into the ELISA plate. Fluorescent signals measured by a micro-plate reader (BioTek EL800, Winooski, VT) were converted into μg/L (S100B) and μg/L (CK-MM) as per standard curve concentrations. The experimenter performing the assay was blinded from subject information.

### Physiological Load Measure

Excess post-exercise oxygen consumption (EPOC) value was used to account for the possible effect of physical exertion. During a bout of exercise, there will be a steep increase in the metabolic demand of the working tissues, leading to a depletion in the volume of oxygen (VO_2_). This physiological mismatch creates an “oxygen debt;” wherein the body is consuming more oxygen than what is available. This oxygen debt remains throughout the exercise bout and is eventually “repaid” upon the cessation of activity, which can be estimated by EPOC (29). EPOC is estimated by VO_2_ levels and duration of the activity: EPOC (ml/kg) = (mean VO_2recovery_ × time _recovery_)–(VO_2baseline_ × time _recovery_) (30). To maximize clinical application, instead of VO_2_, researchers have validated an EPOC measurement using %HR_maximum_, time, and heart rate variability, which yielded a substantial agreement with VO_2_-derived EPOC (range of R^2^: 0.79–0.87) (31, 32). Players in this study wore chest-strap HR monitor from pre-game warm up until approximately 1 h post-game. Peak EPOC values were not utilized because EPOC declines over time and not necessarily in a linear fashion. Thus, the mean EPOC value from each game was assessed for each player using Firstbeat PRO heartbeat analysis software version 1.4.1 and included in analyses.

### Near Point of Convergence

The near point of convergence (NPC) is a well-established assessment of binocular functionality and useful in the evaluation for potential neural injury (33). The subjects in this study were measured at pre-season baseline, pre- and post-games, and post-season using our established protocol (16, 34). The assessment was repeated twice with the mean NPC used for analysis.

### Statistical Analysis

Two-tailed paired *t*-test was performed to examine the difference between pre- and post-game S100B levels for each game, as well as pre- and post-season S100B levels among the players who completed the season. Repeated measures ANOVA was used to evaluate the magnitude of change in S100B level (post minus pre) in 5 games. As we confirmed that the changes in S100B level were similar across 5 games, we combined the data from all the games in the subsequent analyses. We used a multivariate linear regression model to test whether head impact count was associated with the magnitude of the pre-post change in S100B, where the change in S100B was an outcome and head impact was a main predictor. The model was adjusted by physiological effect (EPOC), muscle damage effect (CK-MM), accumulated count of head impacts up to the game (from previous games and practices), pre-season baseline S100B level, and pre-game S100B level. As PLA and PRA were proxies of head impact count, we performed the same analysis using each of them as a main predictor alternative to head impact count. Accumulated PLA or PRA was used as a covariate instead of head impact count in each analysis. *P*-values were adjusted by Bonferroni correction to account for the problem of multiple comparison. To test the difference in goodness of fit between models with and without EPOC and CK-MM, likelihood ratio test (LRT) was performed.

Next, we implemented *k*-means clustering (35) using the three impact kinematic variables (head impact count, PLA, and PRA) and categorized the head impact data into two groups (high vs. low impact group). Because the scale of impact kinematic variables varies with their distributions being right skewed, we used the order statistics of each kinematic variable to keep the sample size of the two groups comparable in the clustering. We then examined whether the pre-post game change in S100B was significantly different between the two groups using two-tailed independent samples *t*-test.

Lastly, Pearson correlation coefficient was conducted to assess a relationship between acute changes in S100B and NPC. All analyses were conducted using statistical software R (version 3.4.1) with package “nlme.”

## Results

### Demographic and Head Impact Kinematics

Two players actively withdrew within the first month, which resulted in 15 players completing the study. There were 24 occasions out of 180 blood draws that we were unable to assess S100B and creatine kinase levels due to subjects being absent during data collection sessions (*n* = 12), a lack of sufficient blood volume (*n* = 7), or hemolysis (*n* = 5). A total of 156 blood samples from 15 players were assessed for S100B and creatine kinase levels and included in the analysis.

A median value of 596 hits, 11,907 *g*, and 1,202,758 rad/s^2^ were recorded from 15 players during pre-season training camp, in-season, and post-season stages. Demographic characteristics and impact kinematics are summarized in Table 1. Please refer to Supplementary Table 1 for the impact data from each game.

**Table 1:** Demographics and head impact kinematics.

**Variables**	**Team (*n* = 15)**
**DEMOGRAPHICS, M (SD)**	
Age, y	16.4 (0.5)
Body mass index, kg/m^2^	28.0 (4.0)
Years football experience	3.13 (1.5)
# of previous concussion	0.47 (0.6)
0	9^*^
1	5^*^
2	1^*^
Race, *n* (%)	
White/Caucasian	10 (66%)
Black/African American	0 (0%)
Asian	3 (20%)
American Indian/Alaska	1 (7%)
Multiracial	1 (7%)
Ethnicity, *n* (%)	
No Latino/Hispanic	10 (66%)
Latino/Hispanic	5 (34%)
**Position**, ***n*** **(%)**	
Linemen (OL, DL)	6 (37.5)
Linebacker, tight end	2 (18.7)
Skill players (WR, DB, RB)	7 (43.8)
**Impact kinematics for season, median (IQR)^†^**	
# of hits	596 (361.5–981.5)
PLA, *g*	11,907 (7,644–21,014)
PRA, rad/s^2^	1,202,758 (637,515–2,100,871)

*IQR, interquartile range; SD, standard deviation*.

* *number of players. OL, offensive lineman; DL, defensive lineman; WR, wide receiver; DB, defensive back; RB, running back; PLA, peak linear acceleration; PRA, peak rotational acceleration*.

† *see Supplementary Table 1 for impact kinematics from individual games*.

### Pattern of Serum S100B Changes Across Study Duration

**Figure 2:**
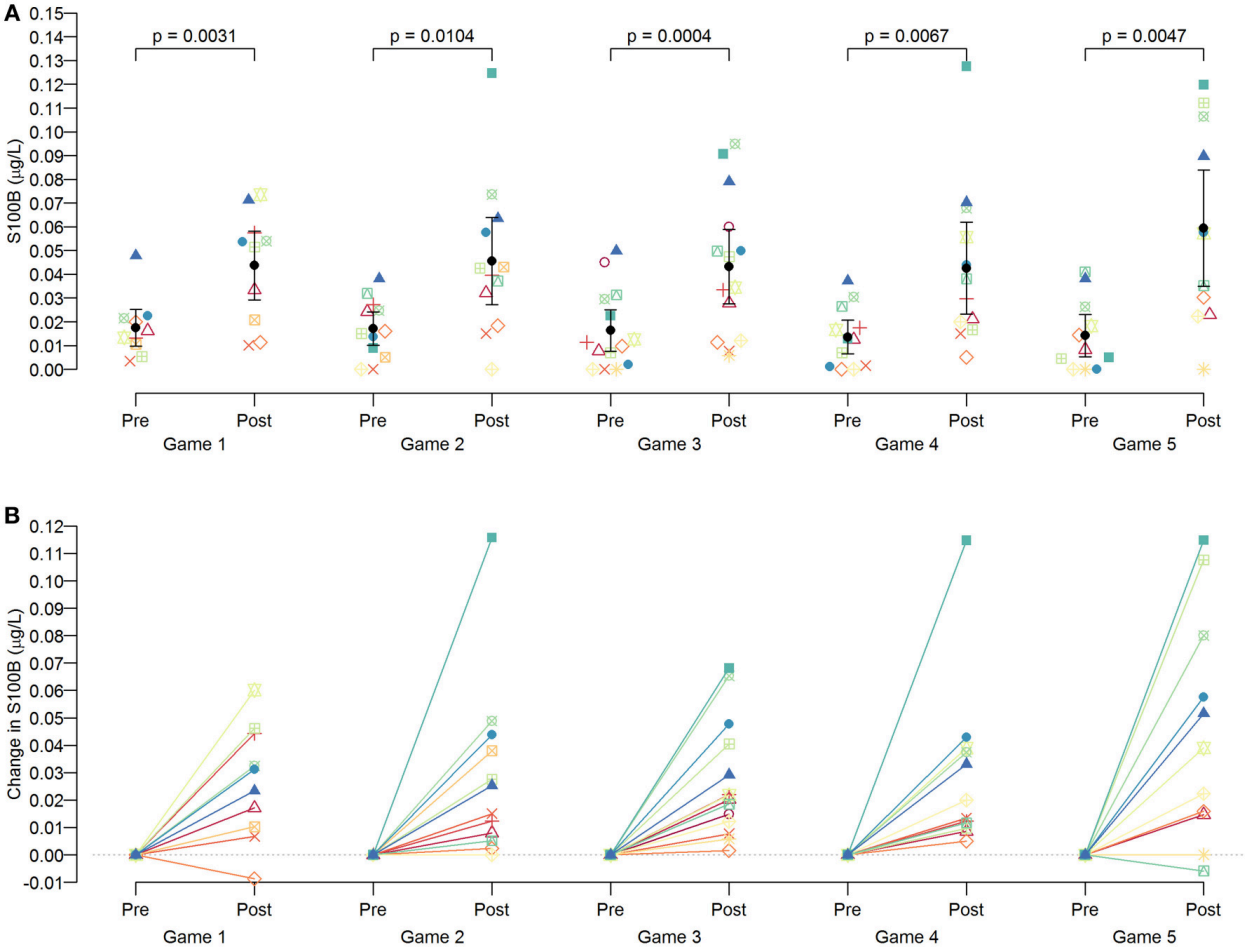
Pattern of S100B changes in players per game. Serum S100B levels were measured at pre-games (4–5 h prior to the game) and post-games (within 1 h from the end of the game). As a group, significant increases (*p* < 0.05 for all games) were observed at post-games compared to the S100B levels of pre-games **(A)**, but an individual pattern showed heterogeneous magnitudes of increase **(B)**.

Serum S100B levels changed significantly, with all post-game S100B levels being significantly higher than the respective pre-game S100B levels (*p* < 0.05 for all games: Figure 2A). Repeated measures ANOVA confirmed that the magnitude of these increases was similar across all games in 15 players [F(4, 34) = 2.25, *p* = 0.08], suggesting that regardless of when the game took place during the season, there was a consistent magnitude of acute increase in serum S100B levels at post-game compared with pre-game. S100B levels were not measured during practices. Nonetheless, serum S100B levels did not reflect on the cumulative subconcussive effects, as there was no significant difference between pre- (0.018 ± 0.013 μg/L) and post-season (0.019 ± 0.019 μg/L) S100B levels (*p* = 0.9581).

### Association of Subconcussive Impact Kinematics on Acute Changes in Serum S100B

As we observed heterogeneous S100B increases at post-games (Figure 2B), multivariate regression models revealed that head impact frequency, PLA, and PRA were positively and significantly associated with the magnitude of the pre-post change in S100B, after Bonferroni correction and adjustment by the covariates. For example, the model estimated that serum S100B would acutely increase by 0.007 μg/L (SE = 0.002) for each additional 10 subconcussive head impacts (Table 2). For the full models with all covariates, please refer to Supplementary Table 2. We performed likelihood ratio test (LRT) to examine whether the physical exertion and muscle damage contributed to the change in S100B. There was no improvement in goodness of fit after factoring EPOC (*p* = 0.252 for hits, *p* = 0.220 for PLA, *p* = 0.227 for PRA) and CK-MM (*p* = 0.838 for hits, *p* = 0.766 for PLA, *p* = 0.803 for PRA) in the models.

**Table 2:** Association of acute S100B increase (post minus pre game: unit in μg/L) with head impact kinematics.

**Predictor variables**	**Estimate (SE)**	***P*-value**	**Adjusted *P*-value**
Head impact in the game (times)	0.007 (0.002)	0.003	0.008
PLA in the game (*g*)	0.0003 (0.0001)	0.002	0.007
PRA in the game (rad/s^2^)	0.000003 (0.000001)	0.005	0.014

*PLA, peak linear acceleration; PRA, peak rotational acceleration; SE, standard error; P-value was adjusted by Bonferroni correction to account for multiple comparison. Please refer to Supplemental Table 2 for the full models with all covariates*.

**Figure 3:**
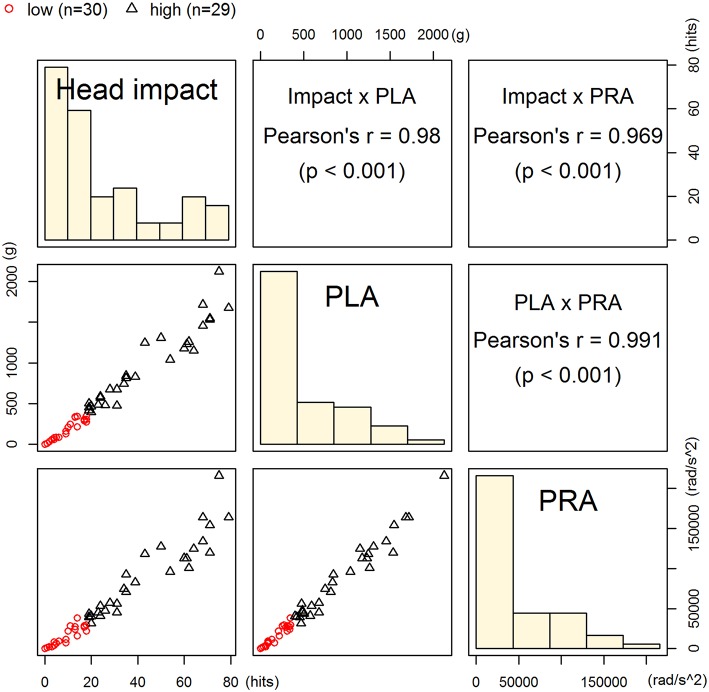
Classification of high- and low-impact groups. Players' data points from 5 games (*n* = 59) were categorized into high (*n* = 30) or low impact group (*n* = 29) using *k*-means clustering on head impact count, peak linear acceleration, and peak rotational acceleration. These kinematic parameters were significantly correlated to one another.

**Figure 4:**
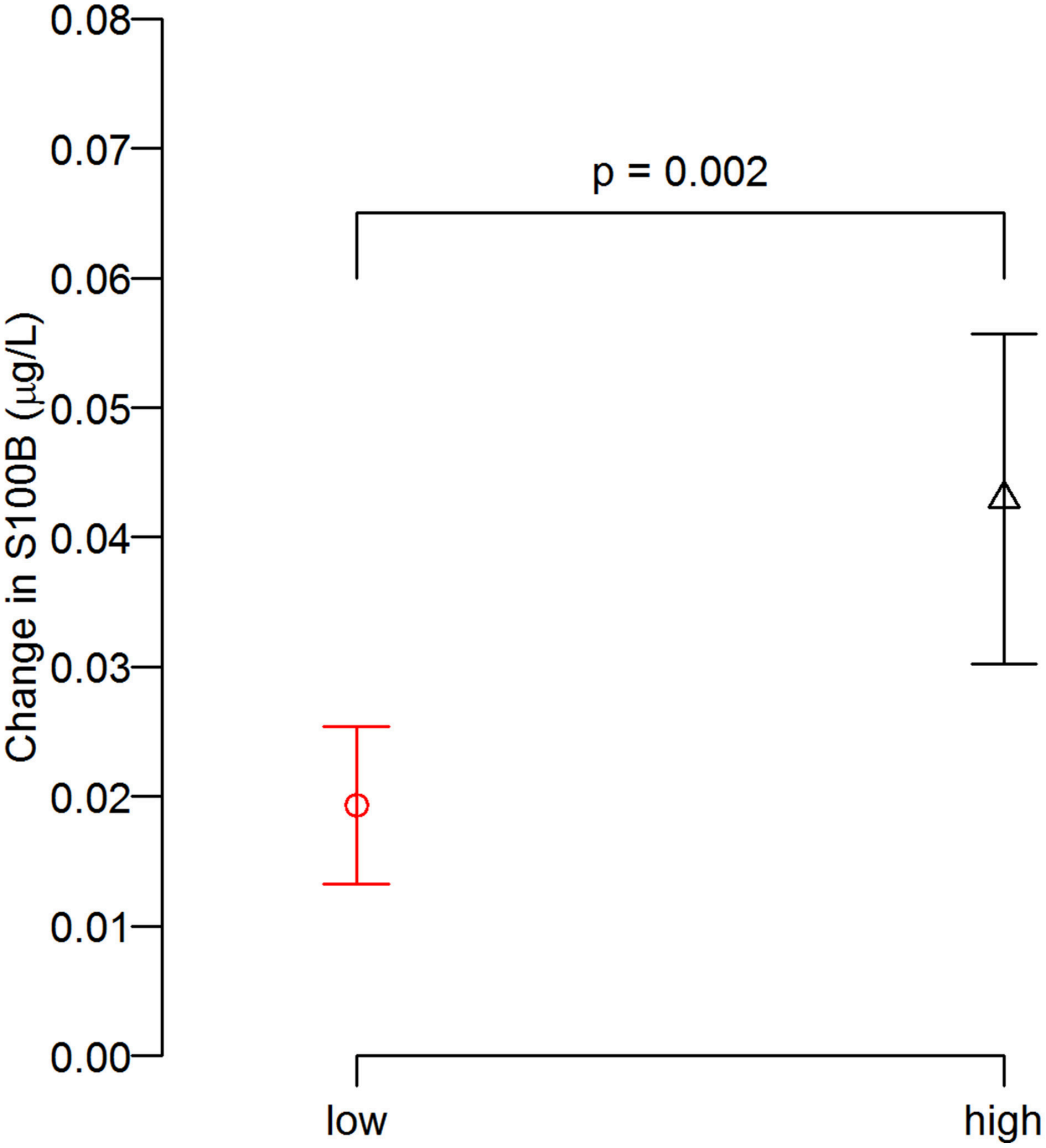
Degree of S100B change (post minus pre) between groups. The post-game S100B increase relative to the pre-game levels in the high impact group was greater than that of low impact group.

Three head impact kinematic variables were highly correlated to one another. As indicated earlier, there were 24 occasions from pre- and post-games that we were unable to acquire S100B values, which equates 12 of 75 possible game data (15 players × 5 games = 75). Additionally, we had four outliers in the impact kinematic data. Collectively, the impact kinematic data from 59 data points (from 15 players) were included in *k*-means cluster analysis, and the data points were categorized in high (*n* = 29) and low impact (*n* = 30) groups (Figure 3). The high impact group showed a significantly greater pre-post change in serum S100B compared to that of the low impact group, t(40.21) = −3.28, *p* = 0.002 (Figure 4).

### Association of the Pre-post Game Changes in S100B Levels With a Behavioral Measure

We used the Pearson correlation coefficient to assess a correlation between the pre-post game changes in S100B levels and NPC. There was an unremarkable relationship between these two variables (*r* = 0.086, *p* = 0.519: Supplemental Figure 1).

## Discussion

To our knowledge, this longitudinal, prospective cohort study is the first clinical study examining the association of serum S100B increase with subconcussive head impact kinematics after adjusting for physical exertion and muscle damage effects in adolescent football players. The data confirmed some of the previous findings and generated critical knowledge about subclinical neurotrauma from repetitive head impacts. First, high school football players experienced a median frequency of 596 subconcussive head impacts in a single season, with some players exceeding 1,000 head impacts. Second, the analysis of 15 football players yielded that serum S100B levels were acutely increased after football games without a concussion. Third, serum S100B did not detect cumulative burden from repetitive subconcussive impacts, possibly due to its short serum half-life (<3 h) (36, 37). Lastly, there was a significant positive association between impact kinematics and increase in S100B levels, and the influence of strenuous exercise and muscle damage on serum S100B levels was negligible.

The novelty of the study includes the assessment of head impact kinematics in adolescent athletes, who are proposed as one of the most crucial age groups whether to prevent or accelerate pathological cascade of neurodegeneration (5, 9, 15, 38). As depicted in Figure 2B, the within-subject increase in S100B levels was similar across 5 games, indicating that some showed a large degree of S100B increase in all games while others consistently showed a modest to moderate increase throughout the study. Our analyses suggest that this pattern attributes to the fact that each player sustained relatively consistent frequency and magnitude of head impacts per game. Since we did not observe significant relationships among S100B, CK-MM, and EPOC, the similar degree of S100B increase in each game is likely due to subconcussive head impact exposure. While player's position may help dissecting the data (39, 40), owing to a lack of sample size and some players played both offense and defense position, we were unable to run such analyses. Nonetheless, our data suggests that the brains of individuals who are prone to high frequencies of head impacts may be experiencing repetitive astrocyte damage, warranting a careful consideration of a safety protocol to regulate how many head impacts are allowed in a season.

As with our data indicate, S100B is well-known for its short plasma half-life due to protease degradation and filtration through the kidney (36, 41, 42), but the intermittent S100B increases induced by subconcussive impacts have the potential to play a key role in long-term neurological consequences. S100B is protective and trophic at low concentrations within the brain parenchyma, whereas it is toxic and pro-apoptotic at high concentrations (10). Specifically, abundant S100B in response to astrocyte damage translocate into an extracellular space and become a ligand for the cell-surface receptor for advanced glycation end products (RAGE) receptors (43), which are expressed on the neuronal plasma membrane. The S100B–RAGE bond induces hyper-phosphorylation of tau protein through the cascade in an order of c-Jun N-terminal kinase (44), Dickkopf-1, and glycogen synthase kinase 3β, which collectively contributing to neurofibrillary tau tangle formation (45) [please refer to the detail cascade depicted in a figure from Kawata et al. (37)]. Cause and effect relationships between a subconcussive impact-induced elevation in S100B, neuronal tau aggregation, and later-onset of CTE require longitudinal investigations, such as an initiative from the CARE consortium (46).

In the current study, as a team, the average serum S100B level from pre-games was 0.016 ± 0.002 μg/L, which was increased by more than 3-fold at post-games (0.05 ± 0.006 μg/L). The magnitude of acute S100B elevation in this study (0.034 μg/L) was slightly less than both Marchi et al. (0.045 μg/L) in a high school football cohort and Kawata et al. (0.058 μg/L) in a college football cohort. These gaps are perhaps due to sampling method difference, serum vs. plasma, practice vs. game, and high school vs. college. Nonetheless, our statistical models incorporated critical covariates to illustrate the association between subconcussive impact kinematics and acute S100B increases. For example, EPOC measures an increased rate of oxygen intake following strenuous activity, often referred as “oxygen debt.” The EPOC accounts for physical exertion during activity and indirectly indicates fat metabolism from the exercise (47). In addition, CK-MM, which modulates intracellular adenosine triphosphate (ATP) levels by catalyzing a phosphoryl group (48), can leave the cell and secretes into the blood stream upon muscle damage from vigorous exercise or contusive trauma (26). Therefore, our data in concert with previous studies suggest that astrocyte damage, as reflected by an increase serum S100B levels, can be triggered by subconcussive head impacts in players who incur impacts continuously.

However, it is important to emphasize that subconcussive neurotrauma is a multi-factorial deficit beyond the changes in cellular and molecular dynamics of astrocytes. Emerging evidence suggests that both acute and chronic exposure to subconcussive head impacts have shown to disrupt neuronal structural integrity (49, 50), neuronal functional connectivity (51–53), vestibular function (54), and neuro-ophthalmologic circuitry (16, 34). Thus, a holistic approach is encouraged when monitoring “at risk” individuals' brain wellbeing.

While the current study employed state-of-art technologies in a well-organized prospective cohort design with 12 data points in a span of 5 months, there were limitations to be noted. A small sample size from a single site inhibits generalizability of the results. However, this is an excellent step for us and others to launch a multi-site longitudinal study to follow players, for example, from freshman to senior in high school and/or college. Repeated venous blood draws can become a safety issue, albeit no issue reported in the current and previous studies (17, 18). While S100B levels did not correlate with the data from a clinical neurological assessment (NPC: Supplemental Figure 1), this study also did not obtain neuroimaging data to confirm neural cell damage and metabolic crisis. However, subconcussive impact-dependent dysregulation in neural network has been suggested and replicated in a number of studies (52, 53, 55, 56), supporting our findings that peripheral serological factor such as S100B may be a productive avenue to evaluate for potential astrocyte injury from subconcussive head impacts. Lastly, while mouthguard sensors have been showing superior performance in detecting accurate kinematic data than other sensors when tightly coupled to the upper dentition (e.g., helmet, headband, skin patch) (57, 58), there are several limitations in the use of mouthguard sensors. Mouthguard sensors including the Vector mouthguard are not permanent device especially for those athletes who chew the mouthguard, which can result in a loose coupling to the upper dentition and may not yield an accurate representation of head movement kinematics. As we acknowledge the importance of tracking the mouthguard fitting, the Vector mouthguard is designed to be re-adjustable, and our research members ensured whether it was fitted tightly on a weekly basis. Also, for future reference, researchers should take into account that some players prefer using their own mouthguards; thus, user compliance can be an issue. Lastly, the Vector mouthguard has not been fully validated in terms of differentiating between relevant and non-relevant impact data, which warrants a future validation study.

In conclusion, evidence begins to unravel the effects of subconcussive head impacts, especially if sustained repeatedly in a narrow time window. S100B is a well-studied blood biomarker with promising utility in reflecting acute brain injury. Our data showed that acute subconcussive head impacts exponentially increased serum S100B levels in an impact frequency and magnitude dependent manner. After accounting for extraneous factors including baseline S100B differences, accumulated head impacts, muscle damage, and physical exertion parameters, our results support the clinical utility of S100B in real-time tracking of acute subconcussive forces and potential brain injury in high school football players.

## Ethics Statement

This study was carried out in accordance with the recommendations of Washington Township Medical Foundation- Institutional Review Board with written informed consent from all subjects. All subjects and their legal guardians gave written informed consent in accordance with the Declaration of Helsinki. The protocol was approved by the Washington Township Medical Foundation.

## Author Contributions

SZ and KK conceptualized and designed the study and the data collection instruments, collected data, drafted the initial manuscript, and reviewed and revised the manuscript. KE designed the study, analyzed the study data, and drafted the initial manuscript, and reviewed and revised the manuscript. ZB and MH validated the study collection instruments, collected data, conducted biomarker experiments, carried out initial analysis, and drafted the initial manuscript. CF and CC designed the data collection instruments, collected data, assisted biomarker experiments, drafted the initial manuscript, and reviewed and revised the manuscript. All authors have read and approved the final manuscript as submitted and agree to be accountable for all aspects of the work.

### Conflict of Interest Statement

The authors declare that the research was conducted in the absence of any commercial or financial relationships that could be construed as a potential conflict of interest.

## Supplementary Material

Supplemental Figure 1 Correlation between acute changes in S100B and near point of convergence. The Pearson correlation coefficient revealed that there was an unremarkable correlation between the two variables.

Supplementary Table 1 Head impact kinematics of different players in each game.

Supplementary Table 2 Multivariate regression model to assess association of acute S100B increase (unit in μg/L) with head impact kinematics after controlled by covariates.
