# Supplementary material for: Association of Increased Serum S100B Levels With High School Football Subconcussive Head Impacts
Source: Front Neurol. 2019 Apr 5;10:327. doi: 10.3389/fneur.2019.00327 (PMC6459945; doi:10.3389/fneur.2019.00327)
Supplement: Supplemental Figure 1 — Correlation between acute changes in S100B and near point of convergence. The Pearson correlation coefficient revealed that there was an unremarkable correlation between the two variables. [file Data_Sheet_1.docx]

Supplemental Figure 1


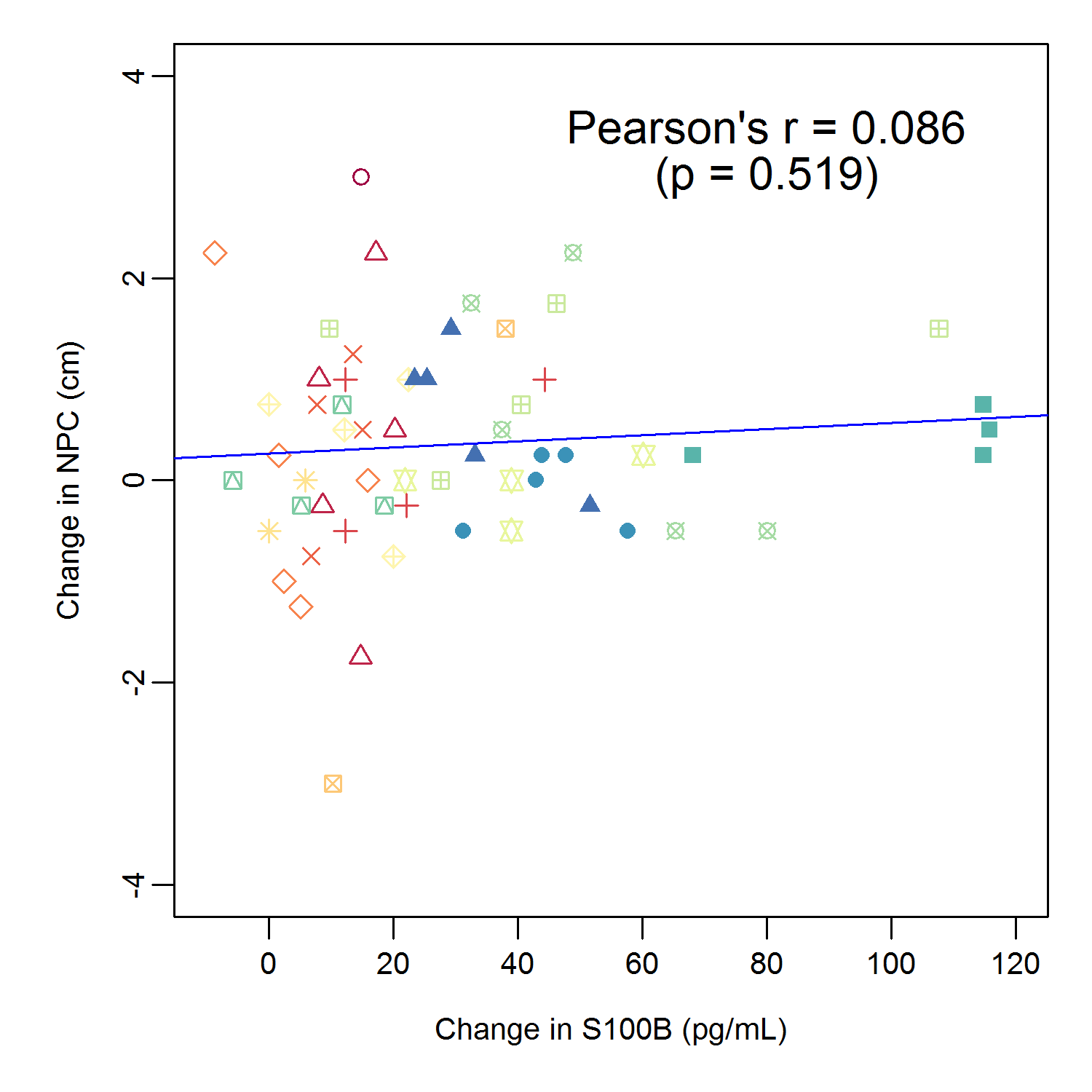


| *Supplementary Table 1. Head impact kinematics of different players in each game* | | | | | |
| --- | --- | --- | --- | --- | --- |
|  | Game 1 | Game 2 | Game 3 | Game 4 | Game 5 |
| Hits | 14.0  (5.0–21.5) | 18.5  (8.5–29.3) | 20.0  (17.5–48.0) | 31.0  (14.0–37.0) | 50.0  (23.5–66.0) |
| PLA (*g*) | 304.0  (85.5–369.3) | 380.5  (203.0–652.0) | 421.0  (300.0–1,037.0) | 482.0  (268.5–822.0) | 1,150.0  (566.5–1,382.0) |
| PRA (rad/s^2^) | 20,181.7  (7,861.5–36,250.1) | 35,320.7  (19,961.2–45,316.0) | 39,711.4  (24,510.4–96,604.2) | 56,045.1  (23,243.0–78,631.0) | 118,255.1  (48,252.6–130,624.0) |
| Note: Impact kinematics data are expressed as median (IQR) per player, per game. IQR, interquartile range. PLA, peak linear acceleration. PRA, peak rotational acceleration. | | | | | |

| *Supplementary Table 2: Multivariate regression model to assess association of acute S100B increase (unit in μg/L) with head impact kinematics after controlled by covariates* | | | | |
| --- | --- | --- | --- | --- |
| **Variables** | **Estimate (SE)** | **P-value** | **Adjusted P-value** |  |
| Intercept | 0.018 (0.012) | 0.145 | 0.434 |  |
| **Head impact in the game (times)** | 0.0.07 (0.0022) | 0.003 | 0.008 |  |
| Cumulative head impact before games (times) | -0.0004 (0.0002) | 0.13 | 0.391 |  |
| Pre-season S100B (μg/L) | -0.0006 (0.005) | 0.902 | 1.000 |  |
| Pre-game S100B (μg/L) | 0.002 (0.004) | 0.564 | 1.000 |  |
| EPOC (ml/kg) | 0.002 (0.002) | 0.236 | 0.707 |  |
| Creatine Kinase (μg/L) | -0.005 (0.01) | 0.585 | 1.000 |  |
|  | **Estimate (SE)** | **P-value** | **Adjusted P-value** |  |
| Intercept | 0.016 (0.012) | 0.175 | 0.524 |  |
| **PLA in the game (g)** | 0.0003 (0.0001) | 0.002 | 0.007 |  |
| Cumulative PLA (g) | -0.00001 (0.00001) | 0.176 | 0.527 |  |
| Pre-season S100B (μg/L) | -0.0009 (0.005) | 0.859 | 1.000 |  |
| Pre-game S100B (μg/L) | 0.003 (0.004) | 0.514 | 1.000 |  |
| EPOC (ml/kg) | 0.003 (0.002) | 0.194 | 0.581 |  |
| Creatine Kinase (μg/L) | -0.007 (0.01) | 0.504 | 1.000 |  |
|  | **Estimate (SE)** | **P-value** | **Adjusted P-value** |  |
| Intercept | 0.018 (0.011) | 0.131 | 0.392 |  |
| **PRA in the game (rad/s^2^)** | 0.000003 (0.000001) | 0.005 | 0.014 |  |
| Cumulative PRA (rad/s^2^) | -0.0000001 (0.0000001) | 0.187 | 0.560 |  |
| Pre-season S100B (μg/L) | -0.001 (0.005) | 0.831 | 1.000 |  |
| Pre-game S100B (μg/L) | 0.002 (0.004) | 0.548 | 1.000 |  |
| EPOC (ml/kg) | 0.003 (0.002) | 0.207 | 0.620 |  |
| Creatine Kinase (μg/L) | -0.006 (0.01) | 0.546 | 1.000 |  |
| Note: Bolded variables are the primary predictor for each model. PLA, peak linear acceleration. PRA, peak rotational acceleration. EPOC, excess post-exercise oxygen consumption. | | | | |
